# Comparing the effect of buprenorphine and methadone in the reduction of methamphetamine craving: a randomized clinical trial

**DOI:** 10.1186/s13063-017-2007-3

**Published:** 2017-06-06

**Authors:** Jamshid Ahmadi, Leila Razeghian Jahromi

**Affiliations:** Substance Abuse Research Center, Shiraz University of Medical Sciences, Shiraz, Iran

**Keywords:** Methadone, Buprenorphine, Methamphetamine withdrawal craving

## Abstract

**Background:**

We sought to test the effectiveness of methadone and buprenorphine in the treatment of methamphetamine withdrawal craving over a 17-day treatment period.

**Methods:**

Patients were randomized into one of two groups. The study sample comprised 40 male subjects dependent on methamphetamine who met criteria of the *Diagnostic and Statistical Manual of Mental Disorders, Fifth Edition*, for methamphetamine dependence and withdrawal and were seeking treatment. Furthermore, they should have a history of daily methamphetamine use for at least 6 months and should have discontinued their use just before starting the protocol. Patients received 40 mg of methadone or 8 mg of buprenorphine per day and were treated in an inpatient psychiatric hospital. We used methamphetamine craving score, negative urine drug screening test (thin-layer chromatography) during the study, and retention in treatment.

**Results:**

All 40 patients completed the study. Both drugs were effective in decreasing methamphetamine craving during methamphetamine withdrawal. Reduction of craving in the buprenorphine group was significantly more than in the methadone group (*P* < 0.05).

**Conclusions:**

The results favor the efficacy and safety of buprenorphine as a short-term treatment for methamphetamine withdrawal craving. We should mention that it is to be expected that craving declines over time without any medication. Therefore, the conclusion may not be that methadone and buprenorphine both reduce the craving. Because buprenorphine is superior to methadone, only buprenorphine surely reduces craving.

**Trial registration:**

Iranian Registry of Clinical Trials identifier: IRCT2015112125160N1. Registered on 4 June 2016.

## Background

Psychiatric disorders have been advancing problems in worldwide [1–4]. Among psychiatric problems, substance use disorders and substance-induced disorders, particularly those involving stimulants, are an increasing global concern [5–7]. In particular, methamphetamine use disorders and methamphetamine-induced psychiatric presentations to hospitals and outpatient centers are becoming increasingly problematic [8–11]. Using amphetamines can cause feelings of euphoria or irritability associated with increases in energy, wakefulness, concentration, and physical activity [7].

Abuse of methamphetamines is common. For example, in the United States, 18 million people over the age of 12 years have tried methamphetamines during their lives [12]. Similarly to other addictions, methamphetamine dependence is a lengthy, relapsing disorder. As part of a comprehensive treatment plan, medications may be required to prevent relapse. Prolonged consumption of methamphetamine can lead to abuse/dependence, aggression, violence, weight loss, impulsivity, decreased appetite, mood lability, poor concentration, hallucinations, delusions, and memory loss [12, 13].

In Iran, methamphetamine previously was illegally imported from other regions of the world (mainly the West), but now it is illegally provided and prepared in “underground” laboratories [10]. Currently, there is no standard of care, particularly medications, for the treatment of methamphetamine craving during methamphetamine withdrawal [7]. Buprenorphine and methadone are opioid medications that are widely used to treat opioid withdrawal symptoms, but, to our knowledge, they have not been used to treat methamphetamine withdrawal symptoms [7].

In this study, we examined buprenorphine and methadone as a way of treating craving during severe methamphetamine withdrawal. We theorized that the biochemistry and mechanisms of methamphetamine and opioid dependence are more or less similar because both drugs involve the endogenous opioid system [7, 10–16].

Substances such as methamphetamine, cocaine, and alcohol activate release of dopamine from cells originating in the brain’s ventral tegmental area. It is a component of a neuronal circuit named the *mesolimbic dopamine system* and is joined to behavioral reward and motivation. Following exposure to alcohol, methamphetamine, or cocaine, dopamine released into the nucleus accumbens and prefrontal cortex strengthens and reinforces alcohol-, methamphetamine-, and cocaine-seeking behaviors [17, 18].

This trial is one of the first studies to provide data obtained by research comparing buprenorphine and methadone in the treatment of methamphetamine craving during methamphetamine withdrawal. The primary goal of this double-blind clinical trial was to test the effectiveness of 8 mg of sublingual buprenorphine daily and 40 mg of oral methadone daily in the treatment of methamphetamine withdrawal craving.

## Methods

### Subjects

Forty unpaid male subjects were recruited in 2016 and were diagnosed with severe methamphetamine dependence and withdrawal on the basis of *Diagnostic and Statistical Manual of Mental Disorders, Fifth Edition* (DSM-5), criteria by a board-certified psychiatrist using the Structured Clinical Interview for DSM-5, Clinical Version. We considered only males because the main psychiatric ward affiliated with the Shiraz University of Medical Sciences admits only male patients.

Prior to each interview, we explained the goals of the study, guaranteed confidentiality, and obtained written informed consent. The interviews and examinations were done on the premises of the treatment hospital because it appeared to be a nonthreatening and proper environment. Family members, friends, or relatives accompanied patients to the hospital. This attendance provided a condition in which we could verify the data and information obtained from the patients.

In addition to meeting DSM-5 criteria for methamphetamine use disorder, subjects required a history of daily methamphetamine use for at least 6 months and discontinuation of their use just before starting the trial. Patients were excluded from the trial if they had a primary diagnosis other than methamphetamine use disorder (dependency on substances other than methamphetamine) or major medical problems (cardiovascular, pulmonary, renal, or gastrointestinal diseases).

All patients provided written informed consent before entering into the trial. The research study was approved and monitored by the ethics committee of Shiraz University of Medical Sciences in adherence to the Declaration of Helsinki’s ethical principles for medical research involving human subjects.

### Randomization

In a double-blind manner, the patients were randomly assigned to one of the two treatment groups: buprenorphine or methadone. We employed a standard randomization procedure generated by computer to obtain a random sample set.

### Procedure

The research staff were precisely trained and included an addiction psychiatrist, general psychiatrist, physician, psychologist, nurse, and statistician. The pills had the same shape and color. The patients and the research team were blinded to the medications for the duration of the trial. The ratings and interviews were performed by an adequately trained physician who was blinded to the medications and side effects. During the trial, no other intervention was allowed.

The principal investigator prepared a visual analogue scale (VAS) and verified it empirically for validity and reliability [10, 14–16]. We used the VAS to assess methamphetamine craving during methamphetamine withdrawal, with scores ranging from 0 to 10 (0 = no craving at all and 10 = severe craving and temptation all the time). Moreover, we trained the subjects precisely and completely about scoring. In addition, a positive urine drug test for methamphetamine (thin-layer chromatography) before the beginning of the protocol and a negative urine drug test twice weekly during the study period were considered.

Consecutive patients were randomly assigned to receive either buprenorphine or methadone. Patients were randomly initiated on either 8 mg of sublingual buprenorphine or 40 mg of oral methadone daily. We followed the subjects for up to 17 days. Effectiveness was evaluated by daily interview and precise assessment of craving by asking the subjects about their experience.

### Statistical analysis

Data analysis was carried out using PASW Statistics version 18 software (SPSS, Chicago, IL, USA). Student’s *t* test or the Mann-Whitney *U* test was used to examine the differences in means. Chi-square analysis or Fisher’s exact test was used to test for differences in frequencies. Repeated measures analysis of variance was used to examine the trends over time. All *P* values were two-sided, and statistical significance was set at the 5% level.

## Results

**Fig. 1:**
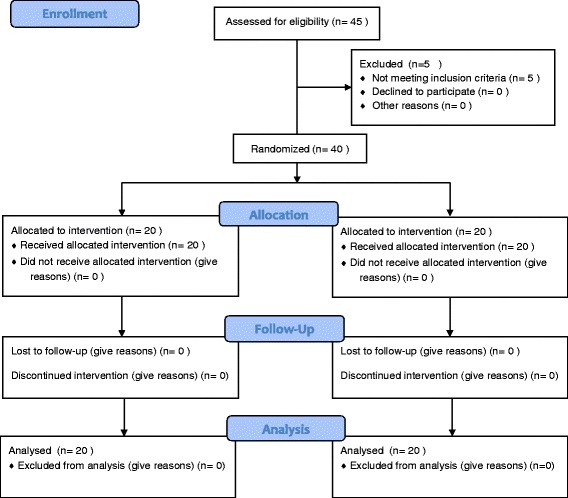
Consolidated Standards of Reporting Trials (CONSORT) flowchart of the patients in this trial

**Fig. 2:**
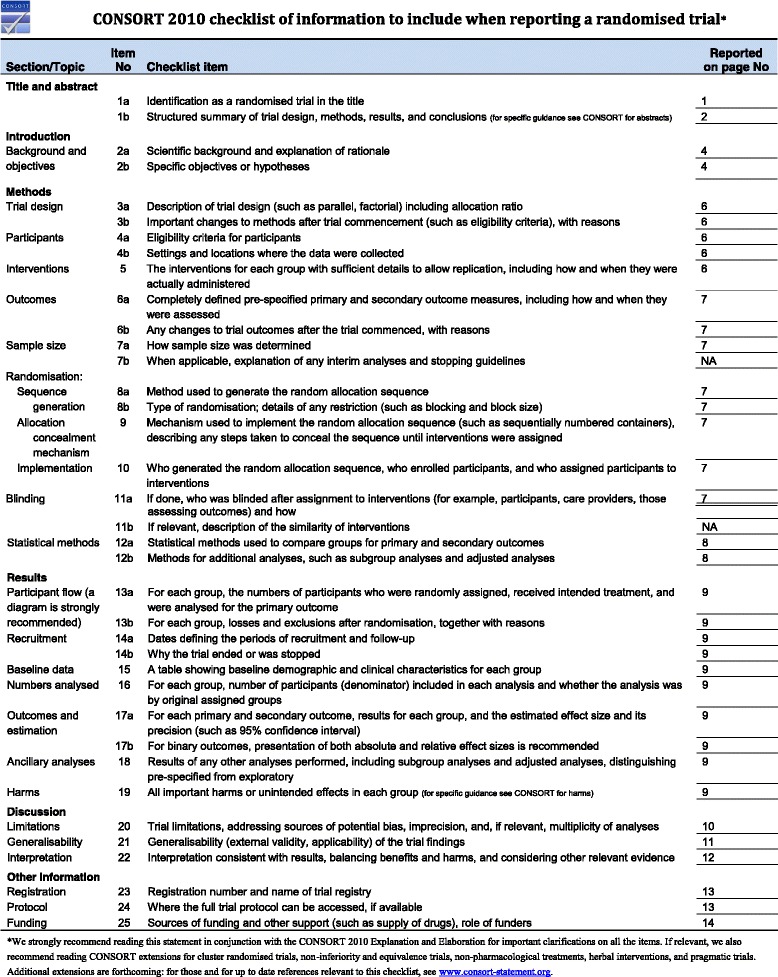
Consolidated Standards of Reporting Trials (CONSORT) 2010 checklist of information to include when reporting a randomized trial

The Consolidated Standards of Reporting Trials (CONSORT) flowchart and checklist of patients in the trial are shown in Figs. 1 and 2. Forty-five patients were screened for this trial. Five patients were excluded because they did not meet the inclusion criteria. Of the 40 patients who were randomly allocated into one of the two groups, 20 patients were assigned to the methadone group and 20 patents were allocated to the buprenorphine group.

All of the 40 patients completed the 17-day trial. Of the 40 patients, 20 (50%) received 8 mg of buprenorphine and 20 (50%) received 40 mg of methadone. Table 1 displays the demographic characteristics of both groups.

**Table 1:** Demographic characteristics of the two study groups

		Methadone (*n* = 20)	Buprenorphine (*n* = 20)	Total	Significance
Age, years, mean ± SD		31.2 ± 9.04	34.35 ± 9.65	32.78 ± 9.37	*t* = 1.065 *df* = 38 *P* = 0.294
Job, *n* (%)	Unemployed	6 (30)	6 (30)	12 (30)	χ^2^ = 0.15 *P* = 0.928
	Self-employed	9 (45)	8 (40)	17 (42.5)	
	Employee	5 (25)	6 (30)	11 (27.5)	
Marital status	Single	9 (45)	10 (50)	19 (47.5)	*P* = 0.64
	Married	11 (55)	10 (50)	21 (52.5)	
Education	Illiterate	5 (25)	5 (25)	10 (25)	*P* = 1
	Middle school	9 (45)	9 (45)	18 (45)	
	High school	4 (20)	4 (20)	8 (20)	
	Higher education	2 (10)	2 (10)	4 (10)	
Income (million Tooman)	<0.5	2 (10)	3 (15.8)	5 (12.5)	*P* = 0.736
	0.5 < 1	16 (80)	12 (63.2)	28 (70)	
	1 < 1.5	1 (5)	3 (15.8)	4 (10)	
	>1.5	1 (5)	1 (5.3)	2 (5)	

There were not any statistically significant differences between the two groups regarding age, education, employment, marital status, or income. According to Table 1, the mean age of 40 methamphetamine dependents was 32.78 years (SD 9.37, range 21–55). The mean ages were 34.35 years (SD 9.65) for the buprenorphine group and 31.2 years (SD 9.04) for the methadone group. Table 2 indicates *t* tests and analysis of variance with repeated measures for craving scores of both groups.

**Table 2:** Independent *t* test and repeated measures analysis of variance for craving mean at 17 days in treatment groups

	Buprenorphine (*n* = 20)	Methadone (*n* = 20)	*t* value	*df*	*P* value	Power
Day 1	7 ± 1.34	7.2 ± 1.28	0.483	38	0.632	0.076
Day 2	6.05 ± 1.76	6.55 ± 1.67	0.921	38	0.363	0.147
Day 3	5.55 ± 1.88	6.3 ± 1.92	1.248	38	0.22	0.23
Day 4	4.6 ± 1.93	5.8 ± 1.88	1.991	38	0.054	0.493
Day 5	4.45 ± 1.76	5.45 ± 2.11	1.625	38	0.112	0.355
Day 6	3.85 ± 1.79	5 ± 2.05	1.891	38	0.066	0.454
Day 7	3.5 ± 1.57	4.6 ± 2.14	1.854	38	0.72	0.438
Day 8	2.9 ± 1.59	4.05 ± 2.16	1.917	38	0.063	0.463
Day 9	2.7 ± 1.56	3.7 ± 2.05	1.734	38	0.091	0.394
Day 10	2.3 ± 1.34	3.45 ± 1.96	2.166	38	0.37	0.558
Day 11	1.9 ± 1.33	3.05 ± 1.79	2.303	38	0.027	0.612
Day 12	1.5 ± 1.32	2.75 ± 1.55	2.746	38	0.009	0.764
Day 13	1.15 ± 1.18	2.45 ± 1.23	3.402	38	0.002	0.914
Day 14	1 ± 1.03	2 ± 1.08	3.008	38	0.005	0.832
Day 15	0.65 ± 0.88	1.6 ± 1.27	–	–	0.18^a^	0.763
Day 16	0.4 ± 0.75	1.4 ± 1.23	–	–	0.11^a^	0.854
Day 17	0.15 ± 0.37	0.8 ± 0.95	–	–	0.35^a^	0.783
*F*	125.572	111.169				
*df*	16	16				
*P* value	0.000	0.000				
Power	1	0.947				
Total of 17 days	2.92 ± 1.189	3.89 ± 1.517	2.251	38	0.03	0.599

^a^ Mann-Whitney *U* test

**Fig. 3:**
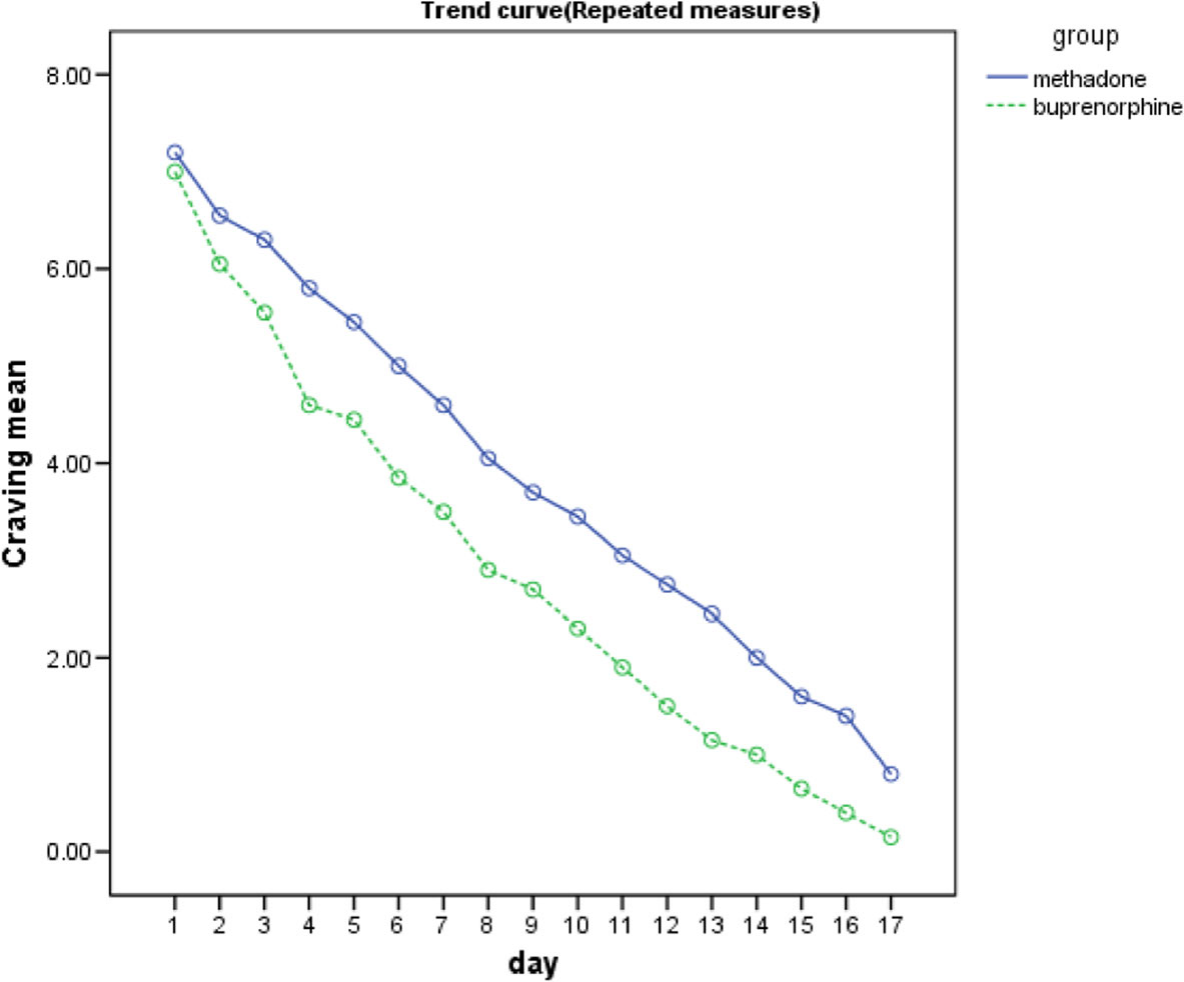
Comparison of mean craving between the two treatment groups

Figure 3 depicts our comparison of the mean craving between the two treatment groups.

On the basis of the data shown in Table 2 and Fig. 3, the methamphetamine craving score was reduced significantly in both the methadone and buprenorphine groups. (repeated measures analysis of variance; buprenorphine *F* = 125.572, *P* = 0.000; methadone *F* = 111.169, *P* = 0.000).

All the patients had positive urine drug test results for methamphetamine at the beginning of the study. Furthermore, all the patients had negative urine drug tests for methamphetamine done twice weekly during the 17-day study interval. During the trial, none of the patients developed significant side effects requiring treatment.

## Discussion

To our knowledge, this is the first study to examine administration of methadone and buprenorphine for the treatment of methamphetamine craving during methamphetamine withdrawal. This study shows that although buprenorphine and methadone are both effective in treating methamphetamine craving during methamphetamine withdrawal, the craving in the buprenorphine group was significantly lower than that in the methadone group starting on the tenth day. Therefore, buprenorphine was more effective than methadone. It is to be expected that craving decreases over time without any medication. Thus, the conclusion cannot be drawn that methadone and buprenorphine both reduce the craving. Because buprenorphine is superior to methadone, only buprenorphine surely reduces the craving.

Patients in both groups did not report any significant side effects. Furthermore, we did not observe any side effects or complications related to buprenorphine or methadone. Besides, the cost considerations seem to be favorable, especially when we study the possibility of administration for outpatients without a need for hospitalization. We suggest these opioids as short-term inpatient treatments to enhance retention or even as long-term maintenance treatment to minimize relapse.

Opioid receptors, mainly the μ opioid receptor, a member of the opioid neuromodulatory system and of the large family of G protein-coupled receptors, are the prominent pharmacological target for the treatment of moderate to severe pain and are of therapeutic value for the management of abuse of methamphetamines, opioids, cannabis, alcohol, and other drugs [19–29]. The mechanism of action by which opioids such as buprenorphine or methadone prevent or decrease methamphetamine craving and dependence is not fully understood; however, there are fundamental and basic interactions between the endogenous opioid neuropeptide systems and dopamine.

Naltrexone, which is an opioid antagonist, reduces and interrupts the interactions between dopamine and endogenous opioid neuropeptide systems [19–21]. We theorized that opioid medications such as buprenorphine can enrich and improve the interactions between dopamine and endogenous opioid neuropeptide systems.

The findings of this research study are supportive of the effect of buprenorphine for the management of methamphetamine craving. There was superiority of buprenorphine compared with methadone (*P* = 0.03). We advise buprenorphine as short-term and inpatient treatment to increase retention or even as long-term maintenance treatment to reduce relapse.

### Limitations of the study

Although we did not have a no-medication control group or a group treated with placebo in addition to the groups treated with buprenorphine and methadone, the fact that the two medications differed significantly in decrease of methamphetamine craving can compensate for this limitation; comparing the mean of craving between buprenorphine and methadone groups, there is a significant difference- (*P* = 0.03). We require a follow-up study to observe what happens when subjects are discharged from a controlled environment. It would be required to specify whether buprenorphine prevents short-term or long-term relapse.

## Conclusions

The outcomes indicated a considerable reduction not only in the craving within each of the two groups but also between the groups. We believe that buprenorphine is a safe, effective, and valuable medication for decreasing methamphetamine craving during methamphetamine withdrawal and more effective than methadone. We recommend consideration of buprenorphine as a treatment for methamphetamine craving during methamphetamine withdrawal. It is to be expected that craving declines over time without any treatment. So, the conclusion cannot be that methadone and buprenorphine both decrease the craving. Because the buprenorphine is superior to methadone, only buprenorphine surely decreases the craving.
